# Comparison of Serum Hepatitis B Virus RNA Levels and Quasispecies Evolution Patterns between Entecavir and Pegylated-Interferon Mono-treatment in Chronic Hepatitis B Patients

**DOI:** 10.1128/JCM.00075-20

**Published:** 2020-08-24

**Authors:** Xiao-qi Yu, Ming-jie Wang, De-min Yu, Pei-zhan Chen, Ming-yu Zhu, Wei Huang, Yue Han, Qi-ming Gong, Xin-xin Zhang

**Affiliations:** a Research Laboratory of Clinical Virology, Ruijin Hospital, Shanghai Jiaotong University School of Medicine, Shanghai, China; b Clinical Research Center, Ruijin Hospital North, Shanghai Jiaotong University School of Medicine, Shanghai, China; c Department of Gastroenterology & Hepatology, Ruijin Hospital North, Shanghai Jiaotong University School of Medicine, Shanghai, China; d Department of Infectious Diseases, Institute of Infectious & Respiratory Diseases, Ruijin Hospital, Shanghai Jiaotong University School of Medicine, Shanghai, China; Cepheid

**Keywords:** hepatitis B virus, HBV, quasispecies, QS, nucleos(t)ide analogue treatment, entecavir, ETV, pegylated interferon, Peg-IFN

## Abstract

Hepatitis B virus (HBV) RNA may independently predict virological and serological response. This study aimed to compare dynamic changes in serum HBV RNA levels and HBV quasispecies evolution patterns between entecavir and pegylated-interferon mono-treatment in chronic hepatitis B patients and to determine the clinical significance during treatment. TaqMan real-time PCR was used for quantitative analysis. HBV RNA levels were retrospectively determined in serial serum samples from 178 chronic hepatitis B patients who received either entecavir or pegylated-interferon treatment. Both serum HBV DNA and RNA quasispecies were analyzed via next-generation sequencing. Receiver operating characteristics (ROC) analysis was performed to evaluate the prediction value of individual biomarkers for hepatitis B e antigen (HBeAg) seroconversion. Patients who received pegylated-interferon treatment showed stronger declines in HBV RNA levels than did those who received entecavir treatment. Serum HBV RNA levels were lower in patients with subsequent HBeAg seroconversion. At baseline, the level of HBV RNA was better than other indicators in predicting HBeAg seroconversion. Moreover, the predictive value of serum HBV RNA levels was better in the entecavir group. Baseline HBV RNA exhibited a significantly higher genetic diversity than HBV DNA and had a significant decline after 4 weeks of entecavir treatment. Higher baseline genetic diversity may result in a better outcome in pegylated-interferon-treated patients. Serum HBV RNA levels showed different decline kinetics, and HBV RNA quasispecies showed different evolution patterns in entecavir and pegylated-interferon mono-treatment. Taken together, serum HBV RNA may serve as a promising biomarker of HBeAg seroconversion in patients during antiviral treatment.

## Abstract (precis)

Hepatitis B virus (HBV) RNA may independently predict virological and serological response. This study aimed to compare dynamic changes in serum HBV RNA levels and HBV quasispecies evolution patterns between entecavir and pegylated-interferon mono-treatment in chronic hepatitis B patients and to determine the clinical significance during treatment. TaqMan real-time PCR was used for quantitative analysis. HBV RNA levels were retrospectively determined in serial serum samples from 178 chronic hepatitis B patients who received either entecavir or pegylated-interferon treatment.

## INTRODUCTION

Hepatitis B virus (HBV) infection is a global health problem (1) and can cause liver cirrhosis or hepatocellular carcinoma (HCC) (2). Because of the persistence of covalently closed circular DNA (cccDNA) as the HBV transcriptional template (3), the majority of chronic hepatitis B (CHB) patients need lifelong therapy to prevent the recurrence of disease activity (4). Despite long-term antiviral treatment, only a few patients can achieve the loss of hepatitis B surface antigen (HBsAg), which is considered to show functional cure (5); therefore, the achievement in noncirrhotic patients of hepatitis B e antigen (HBeAg) seroconversion, which marks partial immune control and long-term virological suppression, may allow treatment withdrawal (6).

Currently, there are two kinds of antiviral treatment to prevent disease progression: nucleos(t)ide analogue (NA) and pegylated interferon (Peg-IFN) (7). Peg-IFN is an immunomodulator and has a weak direct antiviral effect. In contrast, NA blocks reverse transcription, resulting in rapid and effective suppression of HBV DNA to levels below detection, but it does not affect the formation of HBV pregenomic RNA (pgRNA) (8).

Host and viral markers, including alanine aminotransferase (ALT), HBV DNA, HBsAg, and HBeAg, have been confirmed to be associated with response (9) but still have limitations in identification accuracy and prediction value. HBV RNA is a replicative intermediate and may serve as a marker of HBV infection. Recent studies show that HBV RNA detected in serum is mainly encapsidated pregenomic RNA and has potential clinical significance in a number of areas (10), such as predicting treatment response and guiding discontinuation (11). Quasispecies (QS) analysis also indicates that serum HBV RNA is genetically homogenous with intrahepatic HBV RNA (12). However, the kinetics of its decline and the evolution patterns of HBV RNA QS induced by the two different kinds of treatment are still not very clear. In this study, we aimed to compare the decline mode of serum HBV RNA levels and its relation to HBeAg seroconversion and to investigate the evolution patterns of both HBV RNA and HBV DNA quasispecies during the early stage of entecavir (ETV) and Peg-IFN treatments.

## MATERIALS AND METHODS

### CHB patients receiving long-term antiviral treatment.

178 CHB patients were retrospectively enrolled from Ruijin Hospital (Shanghai, China) between 2008 and 2018, among whom 122 were treated with ETV as mono-treatment for >1 year and 56 received 1 year of Peg-IFN treatment. HBeAg-positive patients were divided into two outcome subgroups depending on whether they achieved HBeAg seroconversion within 48 weeks of treatment. Serum samples were collected on the day of therapy initiation (baseline) and at week 4, week 12, week 24, and week 48. All samples were stored at −20°C until assayed. The study was approved by the Ethics Committee of Ruijin Hospital in accordance with the Helsinki Declaration.

### Standard laboratory assessments.

Blood biochemical parameters, including ALT, were measured using an automated chemistry analyzer (Beckman Coulter). Serum HBV DNA levels were quantified using real-time PCR (Shanghai Kehua Bio-Engineering Co., Ltd.) with a lower limit of detection of 500 IU/ml. Quantitative serum HBsAg levels and the presence of HBeAg and anti-HBe antibody were measured using the Abbott Architect immunoassay system (Abbott Laboratories). HBV genotypes were determined by direct sequencing of the pre-S/S gene and comparison with the reference sequences in GenBank (NCBI).

### Extraction of viral nucleic acids.

Total nucleic acids were isolated from 140 μl serum using the TIANamp virus DNA/RNA kit (Tiangen) following the manufacturer’s protocol and were eluted using 60 μl diethyl pyrocarbonate (DEPC)-treated water. An internal control was added to each sample as a control for nucleic acid extraction to exclude false-negative results. Isolated RNA was then treated with DNase I (Ambion).

### Quantification of HBV RNA in serum.

To ensure that no HBV DNA was measured, quantitative PCR (qPCR) was performed in parallel with a digestion control. DNase-treated RNA was reverse transcribed and then amplified using a TaqMan real-time PCR technique with specific HBV primers according to the manufacturer’s instructions (PerkinElmer). The sequences of the primers were as follows (see Fig. S2 in the supplemental material): HBV RNA-FW, 5′-AGACCACCAAATGCCCCT-3′, and HBV RNA-RV, 5′-AGGCGAGGGAGTTCTTCTTCTA-3′. The experiment was performed using the following protocol: 1 cycle at 37°C for 2 min, 50°C for 5 min, 42°C for 20 min, and 94°C for 10 min and 40 cycles at 94°C for 15 s and 62°C for 45 s. The lower limit of detection (LLD) is 200 (2.3 log_10_) copies/ml. For statistical analysis, quantification results below the LLD were adjusted to 2.3 log_10_ copies/ml.

### Next-generation sequencing.

DNase-treated RNA was reverse transcribed using PrimeScript reverse transcription (RT) master mix (TaKaRa Bio, Inc.). The target region of HBV RNA and HBV DNA was amplified using the following barcoded pair of primers (Fig. S2): HBV QS-FW, 5′-AGACGAAGGTCTCAATCGCC-3′ (nucleotides [nt] 2393 to 2412), and HBV QS-RV, 5′-GTTCCCAAGAATATGGTGACCC-3′ (nt 2814 to 2835). The PCR mixture (50 μl) contained 25 μl of PrimeStar HS premix (TaKaRa Bio, Inc.), 1 μl of forward primer (10 μM), 1 μl of reverse primer (10 μM), 5 μl of DNA or cDNA template, and 18 μl of double-distilled water. The PCR cycling conditions were as follows: 95°C for 5 min, 35 cycles at 95°C for 15 s, 56°C for 30 s, and 72°C for 30 s, with a final extension of 72°C for 6 min. Deep sequencing of the PCR products was performed using an Illumina MiSeq platform according to the manufacturer’s 2 × 300-bp paired-end-sequencing protocol.

### QS sequence data analysis.

The nature of the viral population was characterized by viral genetic complexity and diversity using data from the two methods. The QS complexity, known as the Shannon entropy (Sn), was defined as the proportions of different genome sequences in a mutant distribution. Possible values of Sn range from 0 (when all sequences are identical) to 1 (when each sequence is unique). Sn can be calculated with the formula $\text{Sn}=-\sum_{i}\left(p_{i}\text{ ln }p_{i}\right)/\text{ln }N$, where *N* is the total number of clones and *p_i_* is the frequency of each clone in the viral QS population. The QS diversity, defined as the number of mutations that distinguished any two sequences from the population, was evaluated by the mean genetic distance (*d*; also called Hamming distance). Estimation of mean genetic distance was conducted using the Tamura 3-parameter model. Before downstream analysis, error correction was carried out by using previously published software (13, 14). All parameters were calculated using MEGA 7.0 software.

### Statistical analyses.

HBV DNA, HBsAg, HBeAg, and HBV RNA data were log_10_ transformed prior to analyses and expressed as mean (standard deviation). The values were compared by Student’s *t* test or the Mann-Whitney test as appropriate. To assess the distribution of HBV genotypes in different patient groups, a chi-square test (χ^2^) was applied. Logistic regression was used to identify factors associated with HBeAg seroconversion. Receiver operating characteristics (ROC) analysis was performed to evaluate the prediction value in HBeAg seroconversion. The area under the ROC curve (AUROC) was calculated to compare different variables. Optimal cutoff values were obtained from Youden’s index (*J* = sensitivity + specificity − 1). The sensitivity, specificity, positive predictive value, and negative predictive value were calculated at the optimal cutoff value. Graphs were plotted using GraphPad Prism 7.0. Statistical software used included SPSS 24.0 and MedCalc 15.2.2. A two-sided *P* value of less than 0.05 was considered statistically significant.

### Data availability.

The data that support the findings of this study are available from the corresponding author upon reasonable request.

## RESULTS

### Demographic, clinical, and laboratory data.

Study population characteristics are shown in Table 1. Overall, 178 CHB patients were enrolled in this study. The mean age was 37.8 years, and 127 patients were male and 51 female. The HBV genotype distribution was 59 (35%) for genotype B and 110 (65%) for genotype C. Among the patients, 122 were treated with ETV (72 were HBeAg positive) and 56 were treated with Peg-IFN (44 were HBeAg positive). No differences between the two treatment groups were found in HBV genotype or the mean levels of ALT, HBsAg, HBeAg, HBV DNA, or HBV RNA at baseline.

**TABLE 1:** Patient baseline characteristics by treatment group and HBeAg status

Characteristic	Value(s) (mean ± SD or as indicated) for:					*P* value (ETV vs Peg-IFN)
	All patients (*n* = 178)	HBeAg-positive or -negative patients receiving:				
		ETV (*n* = 122)		Peg-IFN (*n* = 56)		
		Positive (*n* = 72)	Negative (*n* = 50)	Positive (*n* = 44)	Negative (*n* = 12)	
No. male/no. female	127/51	54/18	35/15	27/17	11/1	0.011
Age (yr)	37.8 ± 11.6	35.4 ± 8.9	47.8 ± 12.9	30.8 ± 7.4	36.1 ± 5.2	<0.001
ALT (IU/ml)^*a*^	152.7 ± 147.8	174.1 ± 189.7	110.8 ± 85.6	172.5 ± 121.9	146.7 ± 94.8	0.486
No. (%) with HBV genotype:^*b*^						
B	59 (35)	22 (31)	18 (41)	14 (32)	5 (56)	0.863
C	110 (65)	50 (69)	26 (59)	30 (68)	4 (44)	
HBsAg (log_10_ IU/ml)	3.73 ± 0.75	4.16 ± 0.72	3.19 ± 0.44	3.77 ± 0.71	3.23 ± 0.57	0.388
HBeAg (log_10_ S/CO^*d*^)	2.51 ± 0.87	2.59 ± 0.88	NA^*c*^	2.38 ± 0.83	NA	0.217
HBV DNA (log_10_ copies/ml)	6.61 ± 1.37	7.36 ± 1.00	5.54 ± 1.17	6.97 ± 1.03	5.27 ± 1.46	0.996
HBV RNA (log_10_ copies/ml)	6.41 ± 1.86	7.64 ± 1.37	4.79 ± 1.25	6.78 ± 1.39	4.46 ± 1.65	0.529

a Twenty-five patients were without ALT results at baseline.

b Nine HBeAg-negative patients were without genotype data.

c NA, not applicable.

d S/CO, signal-to-cutoff ratio.

### Sequential changes in serum HBV RNA levels during ETV versus Peg-IFN treatment.

**FIG 1:**
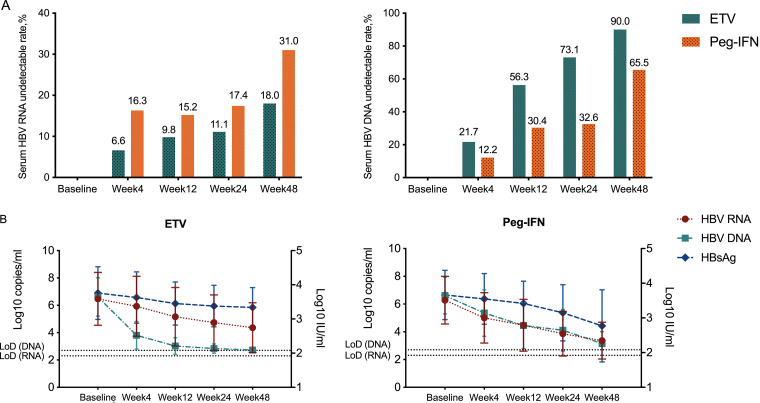
Dynamic changes in serum hepatitis B virus (HBV) RNA, HBV DNA, and hepatitis B surface antigen (HBsAg) levels. (A) Rates of undetectable serum HBV RNA and HBV DNA during antiviral treatment with entecavir (ETV) or pegylated-interferon (Peg-IFN). (B) Sequential changes in serum HBV RNA, HBV DNA, and HBsAg levels during ETV or Peg-IFN treatment. The dotted horizontal lines represent the lower limit of detection (LoD) of HBV RNA (2.3 log_10_) or HBV DNA (2.7 log_10_). Error bars show standard deviations.

Before treatment, serum HBV RNA levels, as well as HBV DNA and HBsAg levels, showed no difference between ETV- and Peg-IFN-treated groups. During 48 weeks of treatment, serum HBV RNA could still be detected even when HBV DNA levels were below the LLD in some patients in both treatment groups (Fig. 1A). In the Peg-IFN treatment group, 65.5% of patients had undetectable HBV DNA levels, and the proportion of patients with HBV RNA levels below the LLD was 31.0% at week 48. Meanwhile, in the ETV treatment group, 90.0% of patients had undetectable HBV DNA levels, but only 18.0% of patients had HBV RNA levels below the LLD at week 48.

ETV showed more powerful suppression of HBV DNA levels than Peg-IFN at all time points during treatment. However, the inhibition of HBV RNA levels was less efficient in ETV-treated patients than in Peg-IFN-treated patients, and thus, HBV RNA levels remained higher than HBV DNA levels at all time points during ETV treatment, whereas HBV DNA levels could be either higher or lower than HBV RNA levels in Peg-IFN-treated patients (Fig. 1B). Peg-IFN induced stronger decreases in HBV RNA levels from baseline to week 4 (1.28 versus 0.54 copies/ml), week 24 (2.42 versus 1.73 copies/ml), and week 48 (2.92 versus 2.11 copies/ml) than did ETV mono-treatment (Fig. S1A in the supplemental material). A significant difference was also found between the reductions of HBsAg levels in the two treatment groups at week 48 posttreatment. HBsAg levels decreased from baseline by only 0.42 log_10_ copies/ml in the ETV treatment group and by 0.89 log_10_ copies/ml in the Peg-IFN treatment group at week 48 (Fig. S1C).

### Serum HBV RNA levels in relation to HBeAg seroconversion.

The mean serum HBV RNA level was 6.41 (1.86) copies/ml at baseline; it was higher in HBeAg-positive patients, at 7.28 (1.48) copies/ml, than in HBeAg-negative patients, where it was 4.75 (1.32) copies/ml (*P* < 0.01). HBV RNA levels declined continuously, though they remained higher in HBeAg-positive patients than in HBeAg-negative patients at all subsequent time points in both treatment groups (*P* < 0.05). The proportions of patients who achieved HBeAg seroconversion were 11/72 (15.3%) in the ETV treatment group and 16/44 (36.3%) in the Peg-IFN treatment group.

**FIG 2:**
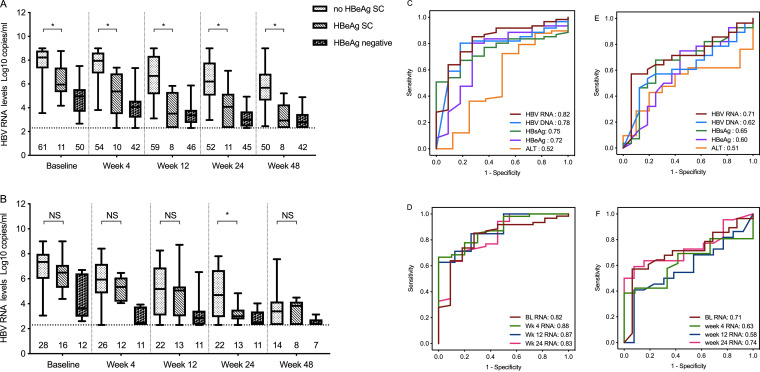
Levels of HBV RNA in relation to HBeAg seroconversion. (A, B) Box plots of serum HBV RNA levels in patients receiving ETV (A) or Peg-IFN (B) treatment according to hepatitis B e antigen seroconversion (HBeAg SC). The number of patients observed at each time point is given below the boxes. The dotted horizontal lines represent the lower limit of detection (2.3 log_10_). Error bars show standard deviations. *, *P* < 0.05; NS, nonsignificant. (C to F) Receiver operating characteristics (ROC) curves describe the performance of the prediction of achievement of HBeAg seroconversion by serum baseline biomarkers and HBV RNA levels during treatment in ETV-treated (C and D) and Peg-IFN-treated (E and F) patients. Numbers in the color key represent the corresponding area under the ROC curve (AUROC) scores. “RNA” refers to serum HBV RNA levels.

ETV-treated patients with subsequent HBeAg seroconversion had significantly lower HBV RNA levels at baseline than those who did not achieve HBeAg seroconversion (Fig. 2A), and stronger declines were observed in the HBeAg seroconversion subgroup than in the no-seroconversion subgroup at all time points. In the Peg-IFN treatment group, patients with subsequent HBeAg seroconversion also had lower HBV RNA levels at baseline and during treatment than those without subsequent seroconversion, but a significant difference was only observed at week 24 (Fig. 2B). In HBeAg-negative patients, a marked decline was also observed in HBV RNA levels during treatment.

### Prediction of HBeAg seroconversion by serum HBV RNA levels.

Lower levels of HBsAg, HBV DNA, and HBV RNA at baseline were correlated with HBeAg seroconversion in all 178 patients by univariate logistic regression, while age, gender, ALT level, and HBV genotype were not associated with HBeAg seroconversion. In multivariate analysis, only the baseline HBV RNA level was an independent predictor of HBeAg seroconversion (Table 2).

**TABLE 2:** Logistic regression analysis of factors associated with HBeAg seroconversion

Parameter	Univariate analysis		Multivariate analysis	
	OR (95% CI)^*a*^	*P* value	OR (95% CI)	*P* value
Age	0.968 (0.92, 1.02)	0.227		
Sex				
Male	Reference value			
Female	1.862 (0.76, 4.58)	0.175		
ALT	1.001 (0.99, 1.00)	0.349		
HBV genotype				
B	Reference value			
C	0.702 (0.28, 1.73)	0.443		
HBsAg	0.450 (0.25, 0.82)	0.009	0.827 (0.33, 2.07)	0.685
HBV DNA	0.634 (0.43, 0.93)	0.023	1.34 (0.68, 2.63)	0.394
HBV RNA	0.524 (0.38, 0.73)	<0.001	0.479 (0.30, 0.77)	0.002

a OR, odds ratio; CI, confidence interval.

We further performed ROC analysis to evaluate the prediction value of factors associated with HBeAg seroconversion. At baseline, the AUROCs of HBV RNA levels were highest in predicting HBeAg seroconversion in both ETV (Fig. 2C) and Peg-IFN (Fig. 2E) groups (AUROC = 0.82 and 0.71, respectively). During treatment, serum HBV RNA levels at week 4 had the best accuracy for distinguishing HBeAg seroconversion patients from no-HBeAg seroconversion patients in the ETV treatment group (AUROC = 0.88) (Fig. 2D), while in the Peg-IFN treatment group, the HBV RNA levels at week 24 best predicted HBeAg seroconversion (AUROC = 0.74) (Fig. 2F). The optimal cutoff values of serum HBV RNA levels and the corresponding sensitivities, specificities, positive predictive values, and negative predictive values were calculated (Table 3). The predictive value of serum HBV RNA levels was better in the ETV treatment group than in the Peg-IFN treatment group.

**TABLE 3:** Predictive performance of serum HBV RNA for HBeAg seroconversion in patients receiving ETV and Peg-IFN

Treatment, time point	Serum HBV RNA optimal cutoff^*a*^	Value (%) for^*b*^:			
		Selectivity	Specificity	PPV	NPV
ETV					
Baseline	6.67	72.73	82.25	47.1	94.5
Wk 4	7.37	100.00	66.67	35.1	100.0
Wk 12	5.84	100.00	62.71	32.6	100.0
Wk 24	5.38	90.91	63.46	31.0	97.5
Peg-IFN					
Baseline	7.23	93.75	57.14	55.6	94.1
Wk 4	6.47	100.00	38.46	42.9	100.0
Wk 12	5.72	92.31	40.91	48.0	90.0
Wk 24	3.77	92.31	59.09	56.3	93.1

a Serum HBV RNA optimal cutoff (log_10_ copies/ml) obtained from Youden's index.

b PPV, positive predictive value; NPV, negative predictive value.

### Serum HBV RNA and DNA QS complexity and diversity.

**FIG 3:**
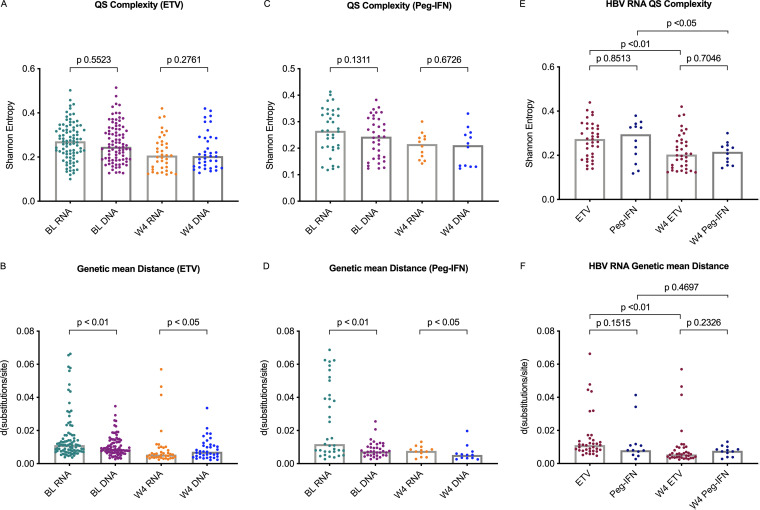
(A to D) HBV RNA quasispecies (QS) evolution patterns. Comparison of hepatitis B virus RNA and DNA quasispecies complexity and diversity values at both baseline (BL) and week 4 (W4) in ETV-treated (A and B) and Peg-IFN-treated (C and D) patients. d, mean genetic distance, also called Hamming distance. (E, F) Dynamic changes in HBV RNA quasispecies genetic characteristics as shown by complexity (E) and diversity (F) between baseline and week 4 in both ETV and Peg-IFN treatment groups. Each patient had paired samples at both baseline and week 4. The top line of the bar indicates the median value.

Because of the low viral load and other technical reasons, we finally obtained high-quality sequencing data for 122 paired HBV RNA and DNA samples at baseline (ETV, 87 paired samples, and Peg-IFN, 35 paired samples). To compare HBV RNA and HBV DNA QS characteristics, both QS complexity and viral genetic distance were calculated. HBV RNA QS complexity and diversity were significantly correlated (*r* = 0.4183, *P* < 0.01), but neither complexity nor diversity was correlated with quantitative HBV RNA levels (Fig. S3). Overall, baseline quasispecies were different between HBV RNA and HBV DNA. (i) Regarding genetic complexity, no significant difference was found between HBV RNA and HBV DNA QS complexity at baseline (Fig. 3A and C). (ii) Regarding genetic diversity, the mean genetic distance of baseline HBV RNA was significantly greater than that of HBV DNA in both treatment groups (Fig. 3B and D).

### HBV RNA QS evolution patterns in ETV versus Peg-IFN treatment groups.

At baseline, HBV RNA QS complexities and mean distances showed no differences between ETV and Peg-IFN treatment groups. To investigate the evolution patterns of HBV QS, both QS complexity and viral genetic distance were also calculated at week 4. Overall, the virus quasispecies were successfully amplified, and matched QS data were obtained in 48 patients at week 4 (ETV, 36 patients, and Peg-IFN, 12 patients). (i) Regarding genetic complexity, the QS complexity of HBV RNA trended to a significant decrease after 4 weeks of treatment in both the ETV and the Peg-IFN treatment group (Fig. 3E). (ii) Regarding genetic diversity, a significant decline in HBV RNA diversity was only observed in the ETV treatment group at week 4 (Fig. 3F). The diversity of HBV RNA was significantly lower than the diversity of HBV DNA at week 4 in the ETV treatment group (Fig. 3B). However, HBV DNA diversity was lower than HBV RNA diversity at week 4 in the Peg-IFN treatment group (Fig. 3D).

**FIG 4:**
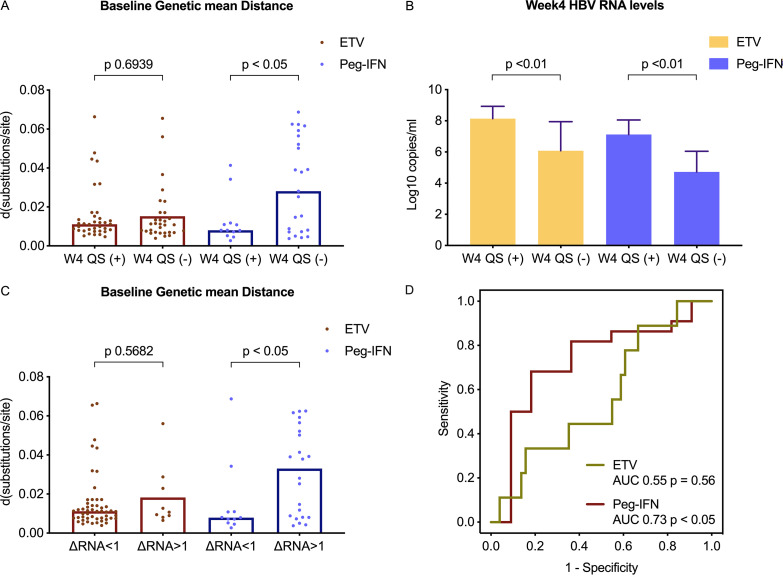
Relationship between HBV RNA quasispecies and clinical outcome. (A) Baseline QS diversity values in different groups. (B) Serum HBV RNA levels at week 4 in different groups. (C) Baseline QS diversity values in patients with an HBV RNA decline either greater than 1 log_10_ copies/ml or less than 1 log_10_ copies/ml after 4 weeks of treatment. (D) Ability of ROC curves of baseline genetic mean distances to predict rapid HBV RNA decline of >1 log_10_ IU/ml after 4 weeks of treatment. d, mean genetic distance, also called Hamming distance; W4 QS (+) or (−), patients with positive or negative HBV RNA QS amplification results, respectively, at week 4; AUC, area under the curve.

Patients in the Peg-IFN treatment group with positive HBV RNA QS amplification results at week 4 had significantly lower baseline mean genetic distances (Fig. 4A); meanwhile, they had significantly higher week 4 HBV RNA levels (Fig. 4B). Therefore, we analyzed the relationship between baseline mean genetic distances and declines in HBV RNA levels. Patients with HBV RNA level declines greater than 1 log_10_ copies/ml after 4 weeks of treatment had greater mean genetic distances, especially in the Peg-IFN treatment group (Fig. 4C). Thus, a greater baseline mean genetic distance may indicate a rapid HBV RNA level decline to >1 log_10_ copies/ml at week 4 posttreatment in the Peg-IFN treatment group (Fig. 4D).

## DISCUSSION

There are five different types of viral RNAs, including the 3.5-kb pregenomic RNA (pgRNA) and precore RNA, 2.4-kb and 2.1-kb HBsAg RNAs, and 0.7-kb HBx RNA (15). However, the origin and the form of serum HBV RNA are still unclear. It is generally accepted that HBV RNA can be released into serum and the major form is enveloped 3.5-kb pregenomic-RNA-containing virions (11, 16). Due to the diversity of RNA forms, different methods have been developed for HBV RNA quantification; hence, the sequence regions used for HBV RNA amplification are also inconsistent (17). Some previous studies used rapid amplification of cDNA ends with PCR (RACE-PCR), which targets the 3′ end of the HBx gene (18–20). In this study, we used a TaqMan real-time technique that is also applied by some other research groups (11, 21–23), with specific primers that target the unique sequence of the 3.5-kb-long HBV RNA. Different amplification regions for full-length or truncated HBV RNA fragments may result in different quantification results, so further evidence is needed to prove consistency among these methods.

Peg-IFN has both immunomodulatory and weak direct antiviral actions to control the virus, while NAs are direct inhibitors of HBV polymerase at the reverse transcription step of HBV replication (24). Previous studies showed the kinetics of changes in HBV RNA levels in NA-based (16) or Peg-IFN-based treatment (20). In the present study, the kinetics of HBV RNA were compared between the two kinds of mono-treatment with different antiviral mechanisms. Because HBV RNA levels are related to the antiviral potency of NAs (25), we only selected patients receiving ETV mono-treatment in the NA cohort to eliminate the potential confounders. Our results showed that serum HBV RNA could still be detected even when HBV DNA levels were below the LLD, and this phenomenon was particularly pronounced in the ETV group. ETV treatment induced weaker declines in HBV RNA levels than Peg-IFN treatment, indicating that, in contrast to its strong suppression of HBV DNA levels, the effect of ETV treatment on HBV RNA levels seems to be limited, since the generation of HBV RNA cannot be inhibited by NA directly.

In accordance with recent findings, we observed a stronger decline in HBV RNA levels in HBeAg seroconversion patients (18), and the decline mode in HBeAg-negative patients was similar to that in HBeAg seroconversion patients, suggesting that serum HBV RNA may reflect the virological and immunological properties of the host. Serum HBV pgRNA may also represent a marker for the persistence and transcription activity of HBV cccDNA (26, 27), and due to the persistence of cccDNA, HBV pgRNA-containing virus can be continuously produced. NAs have no effect on the transcription of HBV cccDNA, and therefore, in HBeAg seroconversion patients treated with ETV, serum HBV RNA levels were significantly lower at baseline and during treatment, indicating better virus control. In contrast, serum HBV RNA levels were not significantly different in patients receiving Peg-IFN with or without subsequent HBeAg seroconversion. Since Peg-IFN can activate the immune system and have a long-term antiviral effect, which leads to the degradation of the viral RNA and the exhaustion of HBV cccDNA, Peg-IFN treatment may also reduce HBV RNA levels in nonresponders to a certain extent; thus, differences between the two subgroups were not sufficient for statistical analysis.

The proportion of patients achieving HBeAg seroconversion was limited even with Peg-IFN treatment (28); therefore, specific indicators or models to predict HBeAg seroconversion are desirable during antiviral treatment. By using antiviral drugs, especially NAs with high antiviral activity, HBV replication can be significantly inhibited and serum HBV DNA levels can be rapidly decreased to below the LLD in the majority of patients. Serum HBsAg levels often remain unchanged, and its production can be derived from either cccDNA or integrated DNA, so it could persist even after prolonged therapy (29); thus, the usefulness of serum HBV DNA and HBsAg levels for prediction is diminished. Some recent findings revealed the utility of serum HBV RNA levels as a novel biomarker to monitor infection and antiviral response. Luo et al. reported that serum HBV RNA levels could predict HBeAg seroconversion during ETV treatment, but their sample size was relatively small (22). Jia et al. found that serum HBV RNA levels might be helpful for predicting HBeAg seroconversion in patients treated with Peg-IFN alone or in combination with adefovir (23). In the present study, we expanded the sample size and compared the prediction value of serum HBV RNA between ETV and Peg-IFN mono-treatment. The results revealed that HBV RNA levels were better predictors for HBeAg seroconversion than baseline HBV DNA and HBsAg levels. Corroborating the previous observations, the absolute HBV RNA levels were superior to the decline from baseline for predicting response (20). The AUROC scores were better in the entecavir treatment group. In this study, a high negative predictive value (>90%) at the optimal cutoff value indicated that HBV RNA levels could help identify nonresponders and prompt the consideration of a combination treatment. Therefore, serum HBV RNA during treatment may serve as a potential new biomarker for monitoring antiviral treatment.

Next-generation sequencing technologies allow massive parallel amplification and detection of individual molecules, which makes them useful in HBV QS studies (30). Previous studies have investigated the early changes in HBV DNA QS during lamivudine treatment (31) and ETV treatment (32) and found that the HBV DNA QS heterogeneity pretreatment may also predict virological outcomes (33). However, the evolution pattern of HBV RNA quasispecies during treatment has not been well characterized, particularly in the early stage. Since the virus reservoir changes dynamically over time and might be constantly replenished by a small amount of ongoing viral replication even under antiviral pressure, it would be interesting to observe the changes in QS patterns of HBV virions from the peripheral blood.

Our results showed that baseline HBV RNA QS diversity was significantly higher than that of HBV DNA, which leads us to speculate that the virus replicates and evolves actively, but only a portion of packaged pgRNA is reversed transcribed into HBV DNA and released into the peripheral blood. When under antiviral pressure, Peg-IFN-treated patients showed QS genetic characteristics quite different from those of ETV-treated patients. It appeared that HBV RNA QS heterogeneity was more likely to be reduced during ETV treatment, and viral QS preserved better replicative fitness in the Peg-IFN group. Higher baseline QS diversity may result in better outcomes in Peg-IFN-treated patients than in ETV-treated patients. The amplified fragment encodes terminal protein (TP), which serves as a primer during reverse transcription and guides the synthesis of minus-strand DNA, and it can also prevent the activation of interferon-induced genes in host cells and inhibit the effect on interferon (34). This may be related to the phenomenon in which the QS diversity of this fragment is more correlated with Peg-IFN treatment response.

In conclusion, we compared the different kinetics of serum HBV RNA levels in the two treatment groups and observed a stronger correlation between HBV RNA levels and subsequent HBeAg seroconversion during treatment. Baseline HBV RNA quasispecies diversity is more relevant to the Peg-IFN treatment response, which leads to the hypothesis that higher HBV RNA QS diversity indicates a relatively efficient host immune response. Overall, these findings will help us to better understand the clinical significance of serum HBV RNA and expand our knowledge of HBV RNA quasispecies evolution patterns in the early stage of ETV and Peg-IFN treatment. We propose that serum HBV RNA may serve as a potential biomarker for predicting HBeAg seroconversion during two different kinds of antiviral treatment.

## Supplementary Material

Supplemental file 1
