# Supplementary material for: Comparison of Serum Hepatitis B Virus RNA Levels and Quasispecies Evolution Patterns between Entecavir and Pegylated-Interferon Mono-treatment in Chronic Hepatitis B Patients
Source: J Clin Microbiol. 2020 Aug 24;58(9):e00075-20. doi: 10.1128/JCM.00075-20 (PMC7448659; doi:10.1128/JCM.00075-20)
Supplement: Supplemental file 1 [file JCM.00075-20-s0001.pdf]

Comparison of serum hepatitis B virus RNA levels and quasispecies evolution patterns between entecavir and pegylated-interferon mono-treatment in chronic hepatitis B patients

Xiao-qi Yu[a], Ming-jie Wang[a], De-min Yu[a], Pei-zhan Chen[b], Ming-yu Zhu[c], Wei Huang[a], Yue Han[a], Qi-ming Gong[d]#, Xin-xin Zhang[a,b]#

Contents:

Supplementary materials consist of 3 figures (Fig S1-S3)

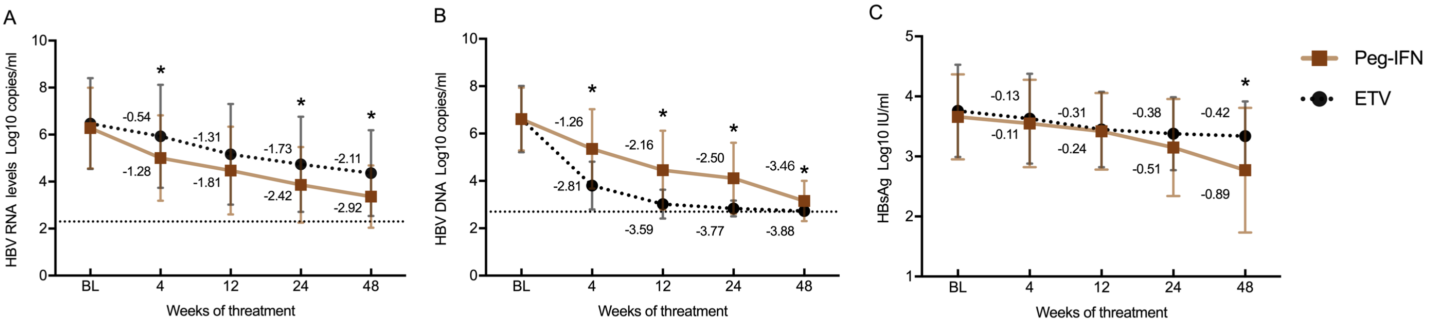

Fig S1: Sequential changes of serum HBV RNA (A), HBV DNA (B) and HBsAg (C) levels between ETV and Peg-IFN treatment groups. Numbers represent mean declines at each time points according to treatment. Statistically differences are labeled. The dotted horizontal line represents the lower limit of detection of HBV RNA (2.3 $\log_{10}$) or HBV DNA (2.7 $\log_{10}$). BL: baseline. HBsAg, hepatitis B surface antigen; HBV, hepatitis B virus; ETV, entecavir; Peg-IFN, pegylated- interferon.

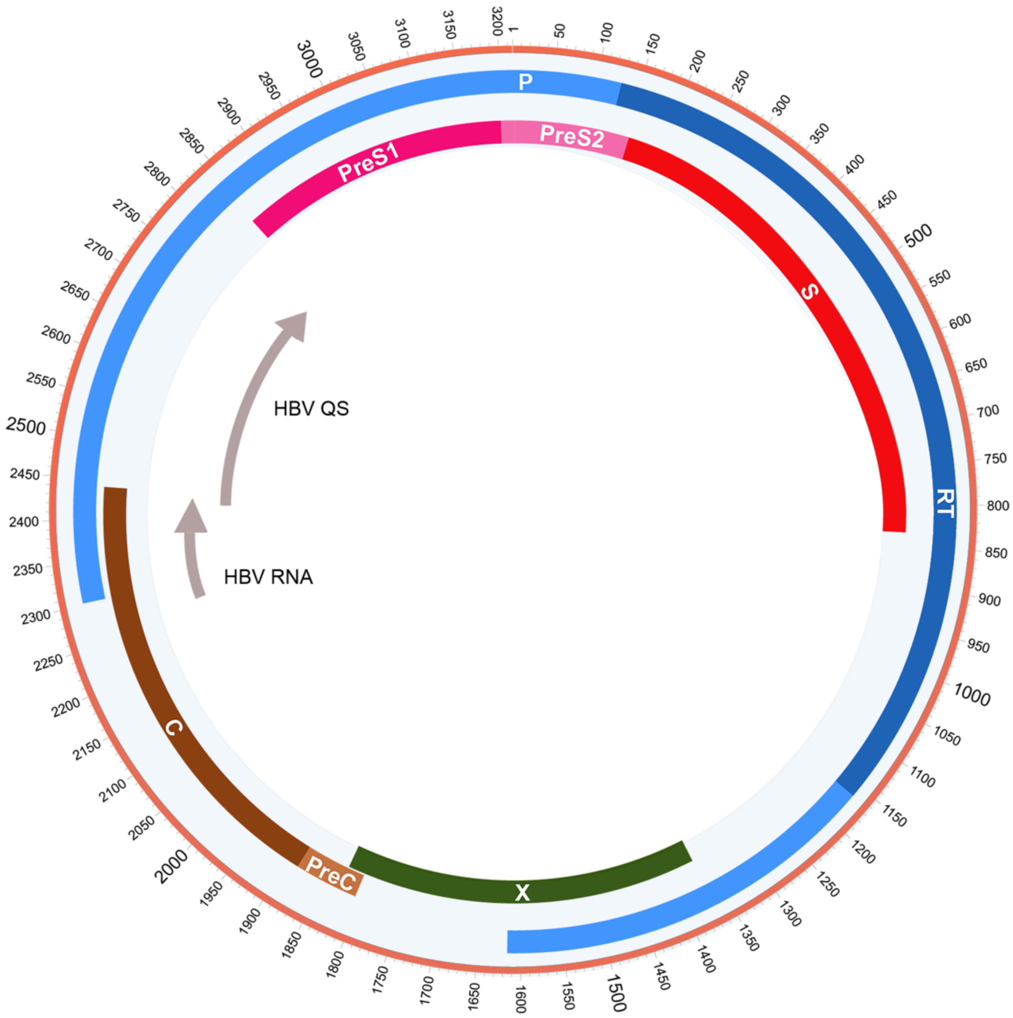

Fig S2: Genome location of HBV RNA quantification (HBV RNA) and HBV quasispecies amplification (HBV QS).

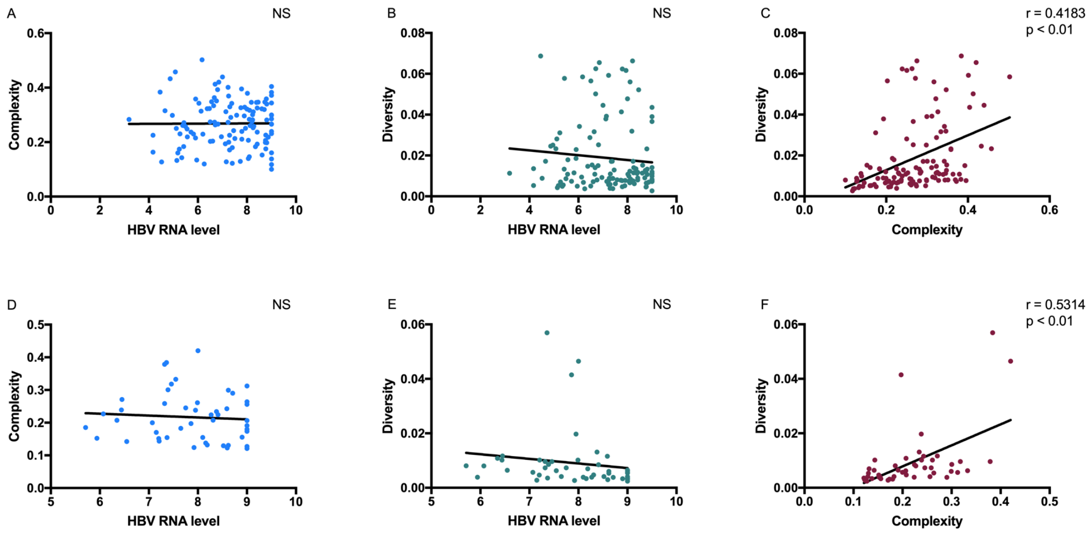

Fig S3: Correlation between HBV RNA quasispecies complexity, diversity, and the level of HBV RNA.
